# The Feasibility and Performance of Total Hip Replacement Prediction Deep Learning Algorithm with Real World Data

**DOI:** 10.3390/bioengineering10040458

**Published:** 2023-04-09

**Authors:** Chih-Chi Chen, Jen-Fu Huang, Wei-Cheng Lin, Chi-Tung Cheng, Shann-Ching Chen, Chih-Yuan Fu, Mel S. Lee, Chien-Hung Liao, Chia-Ying Chung

**Affiliations:** 1 Department of Physical Medicine and Rehabilitation, Chang Gung Memorial Hospital, Chang Gung University, Linkou, Taoyuan 33328, Taiwan; 2 Department of Trauma and Emergency Surgery, Chang Gung Memorial Hospital, Chang Gung University, Linkou, Taoyuan 33328, Taiwan; 3 Department of Electrical Engineering, Chang Gung University, Taoyuan 33302, Taiwan; 4 Department of Orthopaedic Surgery, Pao-Chien Hospital, Pingtung 90078, Taiwan

**Keywords:** total hip replacement, deep learning, artificial intelligence, real world data

## Abstract

(1) Background: Hip degenerative disorder is a common geriatric disease is the main causes to lead to total hip replacement (THR). The surgical timing of THR is crucial for post-operative recovery. Deep learning (DL) algorithms can be used to detect anomalies in medical images and predict the need for THR. The real world data (RWD) were used to validate the artificial intelligence and DL algorithm in medicine but there was no previous study to prove its function in THR prediction. (2) Methods: We designed a sequential two-stage hip replacement prediction deep learning algorithm to identify the possibility of THR in three months of hip joints by plain pelvic radiography (PXR). We also collected RWD to validate the performance of this algorithm. (3) Results: The RWD totally included 3766 PXRs from 2018 to 2019. The overall accuracy of the algorithm was 0.9633; sensitivity was 0.9450; specificity was 1.000 and the precision was 1.000. The negative predictive value was 0.9009, the false negative rate was 0.0550, and the F1 score was 0.9717. The area under curve was 0.972 with 95% confidence interval from 0.953 to 0.987. (4) Conclusions: In summary, this DL algorithm can provide an accurate and reliable method for detecting hip degeneration and predicting the need for further THR. RWD offered an alternative support of the algorithm and validated its function to save time and cost.

## 1. Introduction

Hip joints are the essential weight-bearing joints to connect the trunk and lower extremities. They play a crucial role in ambulances and core stability [1,2]. Once dysfunction of the hip joints occurs, the quality of life and life span of individuals can be impacted. The hip disorders include congenital disorders, degenerative disease, avascular necrosis and fracture, which are the main causes to lead to total hip arthroplasty [3,4,5,6]. Non-surgical treatments are available to reduce pain and improve hip mobility [7,8]. However, if non-surgical treatments fail to provide relief, total hip replacement (THR) may be recommended. The optimizing timing to receiving THR can result in the most easily recovered from decreasing ambulance and activity ability [9,10]. The timing of surgery is also an important consideration, as potential benefits of surgery include increased mobility, reduced pain, and improved quality of life. Various parameters are used for clinical judgment of further surgical intervention for degenerative hip disorder. The decision to undergo total hip replacement is typically based on a combination of factors, including intractable pain, the severity of limitations in mobility, the severity of damage to the hip joint indicated by medical images, the patient’s age and overall health, and medical history [11,12,13]. Current evidence suggests that the combination of all these factors and the patient’s expectations and willingness to undergo the surgery and rehabilitation process will also be taken into account. For primary care physicians, the timing of referral for orthopedic consultation and specialist management is critical, as this can affect further surgical timing. Plain pelvic films (PXR) are the most essential and prevalent tool used as primary surveys for patients with hip disorders [14,15]. They can provide information about the severity of the damage to the hip joint and assist primary doctors in making therapeutic decisions. However, reading PXR films requires experience and familiarity, and sometimes, misdiagnosis leading to postponed referrals can occur. Therefore, it is still challenging to use PXRs only for THR decision.

Digital medical imaging systems offer not only instant remote access, but also the possibility of computer-aided diagnosis. Deep learning (DL) algorithms can be used as computational tools to automatically detect anomalies in medical images [16,17,18]. The use of deep learning models in medical imaging has potential to improve the accuracy and reduce the time and cost of medical imaging analysis [19,20]. It can also be used to identify and classify lesions, detect signs of disease, and predict patient prognosis [21,22,23,24,25]. For the musculoskeletal system, there are several investigative tools such as computed tomography, magnetic resonance imaging, and plain radiographic films. Radiographic films are essential tools that can detect abnormalities in bony structures. Several algorithms were developed to detect skeletal abnormalities in plain films [26,27,28]. From acute traumatic fracture, congenital disorder, oncologic lesion to degenerative disease, there was evidence to support the DL algorithm that can help the clinical doctors to detect the lesion accurately and efficiently [29,30,31,32]. For degenerative disorders, there are also several references that provide significant evidence which DL can offer proper support for clinical doctors. Although these algorithms offer different advantages, there are no algorithms applied to predict the necessary for further THR.

The quality and quantity of training data are essential in training DL algorithms. However, unlike other sectors, collecting medical images on a large scale is challenging. As a result, the datasets used to train and validate DL algorithms were a significant hurdle in integrating medical artificial intelligence (AI) into clinical applications. To address this issue, the real world data (RWD), including patient health status, and health care delivery data that are collected periodically from a variety of sources [33,34] are used to validate the functionality of AI models as an alternative to randomized clinical trials. Increased data support RWD can play a role in DL model validation [35]. Additionally, the RWD announced by regulatory agencies could become the standard validation of DL models prior to market approval [36,37,38]. With the increased accessibility to RWD and the rapid development of deep learning technology, there is great interest in using RWD to increase the efficiency of clinical research and discovery, and to bridge the evidence gap between clinical research and practice [39,40]. DL algorithms can be used to detect changes in patient status over time, particularly for degenerative diseases such as osteoarthritis [41]. Previous experience in applying DL to detect degenerative disorders of the knee and hip was reported [42,43,44]. However, it is difficult to predict the need for further treatment [45,46], which might be helpful for clinical practice. In advance, there was no DL algorithm applied on the THR decisions on hip joints with RWD validation.

In this study, we developed a DL algorithm to predict the necessity of THR within three months and applied RWD to validate the feasibility and clinical performance of this algorithm. By using RWD, the DL algorithm was tested in a more realistic setting, allowing for a more accurate assessment of its clinical performance.

## 2. Materials and Methods

### 2.1. The Data Source and Label

We collected the anteroposterior (AP) pelvic radiographs and operative information of all patients who underwent joint replacement surgery of hips at our hospital from August 2008 to December 2017. Finally, the dataset consisted of 3013 pairs of radiographs before and after total hip replacement surgery and 1630 non-surgical cases from the emergency room without undergoing hip surgery within three months. The study was approved by the Instructional Review Board of Chang Gung memorial hospital with no.201801784B0. We excluded the images of hip fractures, pediatric skeletal images, and poor image quality. The image included foreign body rather than hip and femoral prosthesis were also excluded. The final dataset consisted of 4854 hips joints by using 4643 weight-bearing anterior-posterior PXRs from 4643 participants.

### 2.2. Algorithm Design

**Figure 1:**
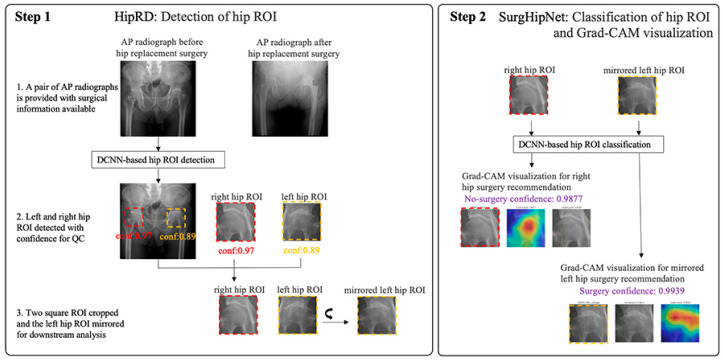
Overview of the proposed two-step classification approach. (**Step 1**): HipRD system detects hip ROI in the provided AP pelvic radiograph. (**Step 2**): SurgHipNet classifies hip ROI and provides Grad-CAM visualization for surgery recommendation. ROI: Region of interest.

We designed a sequential two-stage hip replacement prediction framework to identify the possibility of THR in three months of hip regions of interest (ROI): HipRD [47] and SurgHipNet [48]. The first step was a localization model to identify hip ROI in the provided PXR and cropped ROI from the surrounding background to simplify further processing, avoid noise from other organs, and reduce computational power. The second step was a classification model to distinguish between necessary and unnecessary for THR of imputed ROIs using a classification-based neural network and Grad-CAM heatmap visualization. Localization and classification models were jointly as a pipeline. The workflow of the algorithm is shown in Figure 1.

### 2.3. The Hip Localization

For hip localization training, an automatic practical framework was utilized to detect the ROI of hip joints in each PXRs. The detailed framework development method was described in a previous study [47]. We placed a bounding box at the center of the femoral head to detect the ROIs. All the ROIs in the dataset were visually reviewed by one physician with 15 years of clinical experience to prevent miss-segmentation. All ROIs were initially labeled as THR or no THR according to the surgical reports

### 2.4. The Total Hip Replacement Classification and Visualization

The cropped images were used to develop the SurgHipNet classification model which was utilized by the ResNet-101 network pre-trained on ImageNet and hip joint images from Osteoarthritis Initiative. (https://nda.nih.gov/oai, accessed on 5 May 2018). We modified the block architecture to improve the interpretability of the reasoning process of the learned network. The input hip ROI was resized to 224 × 224 pixels with an 8-bit grayscale color and the output of the model was the probability of THR. We applied fastai augmentation on the data during training with. The detailed framework development method was described in a previous study [48]. During the trained model inference process, for those ROIs to predict THR, we applied the gradient-weighted class activation mapping (Grad- CAM) to determine whether the model correctly focused on the pathologic area of hip joint.

### 2.5. The Real-World Data from 2018 to 2019

There were numerous sources of RWD, such as electronic health records, registry data, claims data, patient-reported outcome data, and data collected from wearables. [39] In this study, we used RWD collected from registries: the Chang Gung Research Registry dataset (CGRD) and Chang Gung Trauma Registries (CGTR). The CGRD consisted of all inpatient data collected from the electronic health records of all patients who visited Chang Gung Memorial hospital. It collected routine care information, including laboratory data, operative reports, perioperative records, summaries of hospitalization and outpatient service, emergency visit records, medical images, examination reports, and claim data. The CGTR was the registry for patients suffering from trauma and visiting Chang Gung Memorial hospital. It included all details related to the injuries. Compared with CGRD, CGTR included specific grades and scores for trauma evaluation, such as the injury severity score (ISS) and abbreviated injury scale (AIS). Moreover, examinations and images were re-evaluated by registers, so there were some injury details in the CGTR.

For the THR group, we extracted the patient’s list from CGRD using operative codes “Total hip replacement”, “Revision total hip replacement”, and “Arthroplasty of hip joint” from January 2018 to December 2019 at a medical center in northern Taiwan. After identifying the THR group, we collected all available preoperative radiographs (PXRs) within three months before THR. We excluded patients who had no PXR three months before surgery, had a fracture, and were younger than 18 years old. All preoperative PXRs were included. We also collected patients from CGRD who visited the emergency department due to traumatic etiology and did not receive hip surgery within three months from January 2018 to December 2019 as non-THR group candidates. Then, we included these candidates who had PXR during this period and extracted the PXRs as non-THR images. As a large dataset, CGRD is noisy, heterogeneous, and unstructured. To avoid missing or overestimating the data, we used CGTR to ensure that all the patients we needed were included in this RWD. We excluded the patients suffering from a fracture from this cohort. Once the PXRs were extracted, we divided them into the THR and non-THR groups, and we then performed deidentification to protect patients’ privacy under ethical guidance.

To better judge the accuracy of the model, we distinguished the ROIs and performed random sampling according to the feature ratio of the predicted grouping under the coding flow. We ensured that the data distribution of the sample was representative of the population to avoid misjudging the accuracy of the model due to sampling errors. In random sampling, due to the significant difference in the proportion of predicted grouping features, we started with the smallest proportion and rounded off the first digit after the decimal point. We also set the Seed random option when selecting data, so that the same data could be randomly sampled according to the random number Seed during random sampling, allowing for repeated verification. We randomly selected 200 PXRs from the THR group and 100 PXRs from the non-THR group to enter SurgHipNet and obtain predictions of the need for surgery for subsequent statistical analysis. Additionally, the model provided a heatmap to help physicians identify lesions and pathological sites.

### 2.6. Statistical Analysis and Software

The developmental and testing process was performed on a workstation with the operating system with Intel (R) Core (TM) i9-10900X CPU @ 3.70 GHz, 96 GB RAM, and one Nvidia Tesla V100 GPU with an Ubuntu 16.04 operative system. The whole pipeline was developed with PyTorch v0.4 and fastai API 2020 implementation and CUDA 9.0. The image labeling process was performed on the self-code toolkit.

Statistical analysis was performed using R 3.6.3 and the Python library scikit-learn [49]. We reported overall accuracy, sensitivity, specificity, negative predictive value (NPV), F1 metric, receiver operating characteristic (ROC) curve, and area under ROC curve (AUC) with 95% confidence interval (CI).

## 3. Results

### 3.1. The Patient’s Distribution of Training Dataset for SurgHipNet and the Performance of SurgHipNet in Testing Dataset

We included 3013 cases in the THR group and 1630 cases in the non-THR group. The mean age of the THR group was 63.1 + 15.7 years old (range: 18–102), which was older than the non-THR group (44.9 + 20.5, range 18–88, *p* < 0.001). The percentage of male gender in the THR group was 40.1% lower than the non-THR group at 68.2% with statistical significance (*p* < 0.001). The algorithms SurghipNet were trained with 3903 hip ROIs which consisted of 924 ROIs before and after THR which was including 205 lower-grade avascular necrosis (AVN)(below grade 3), 516 high-grade AVN (above grade 3), 41 lower-grade osteoarthritis (OA) (below grade 3), 155 high-grade OA (above grade 3), and 17 other etiology. The clinical grading was assigned using a Croft score [50] for OA and Steinberg staging for AVN [51]. A total of 2979 non-surgical ROIs, including 2246 normal cases and 733 lower-grade OA and AVN, from patients in the emergency room without surgery within three months were obtained from the 2008–2017dataset.

The performance was evaluated by testing sets with 475 hip ROIs. By splitting data validation, the overall accuracy, sensitivity, specificity, and AUC were 0.977, 0.9200, 0.992, and 0.994 (95% CI:0.990–0.998) [48].

### 3.2. The 2018–2019 RWD Dataset Distribution

To perform RWD dataset validation, we collected patient data from 2018 and 2019, which were different from the original training data. Our dataset was composed of 1994 patients who underwent THR at CGMH during the specified period. We then applied exclusion criteria to remove patients with fractures, pediatric patients, and those who did not have PXR taken within three months prior to their THR. After these exclusions, we included a total of 744 patients in the final THR group. In addition to the THR group, we included a non-THR group that was composed of 3055 PXR images from patients who did not undergo THR during the same time frame. We again applied the same exclusion criteria to exclude patients. After exclusions, we had a total of 3022 PXRs in the non-THR group. It was important to have a large and diverse dataset that included both THR and non-THR images to validate SurgHipNet. All of the images were de-identified to ensure patient privacy and were then included in RWD. We randomly selected 200 ROIs from the THR group and 100 ROIs from the non-THR group to validate the final performance of SurgHipNet.

### 3.3. Performance of SurgHipNet on RWD Dataset

**Figure 2:**
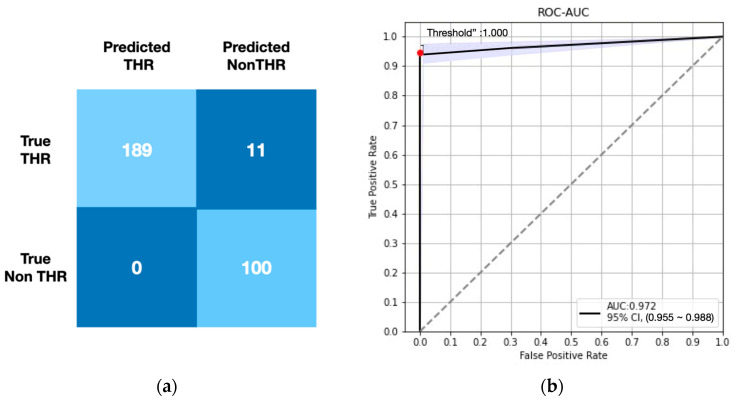
The confusion matrix and ROC Curve of SurgHipNet in real-world dataset. (**a**) The confusion matrix. (**b**) The ROC curve with area under curve. ROC: Receiver operating characteristic.

The performance of SurgHipNet on the RWD dataset was listed in Table 1 and the confusion matrix as Figure 2a. The overall accuracy was 0.963; sensitivity was 0.945; specificity was 1.000, and the precision was 1.000. The negative predictive value was 0.901; the false negative rate was 0.055. Finally, an F1 score of 0.9717 was a harmonic mean of precision and sensitivity and provided a balance between the two metrics. The ROC curve is presented in Figure 2b and the AUC was 0.972 with 95% CI from 0.953 to 0.987. These results suggest that SurgHipNet is a highly accurate model for predicting whether a patient needs to have THR or not.

## 4. Discussion

In the current study, we validated the algorithm SurgHipNet with RWD and the performance was satisfied with high sensitivity (94.5%), specificity (100%), and accuracy (96.3%) (F1 score: 0.97). Till now, we still encounter the hip degenerative disorder daily, and the necessity of employing THR or not is still a critical issue. For primary doctors, with the assistance of the SurgHipNet, we can make the prediction of THR with convincing assistance and arrange further transfers earlier. This project will benefit the geriatric population with hip degenerative disorder. Our DL model will help the primary physicians to determine the severity of the disease and provide the best timing for surgical transferring. In this study, we focused not only on the feasibility and performance of the algorithm but also used the RWD dataset as a validation set to solidify the performance of this model in the proper way. Overall, the results of our study demonstrate the potential of our algorithm as a reliable tool for detecting hip degeneration and predicting the need for THR.

DL allows the computer to learn from iterations without programming and the prediction rate is highly accurate. Although DL was used to predict surgical outcome and trauma risk [46,52], there is limited evidence to support DL used in predicting hip THR. To our knowledge, this is the first study demonstrating that a DL algorithm can predict THR on PXR with satisfying accuracy. The DL model will assist the primary physicians in determining the severity of the disease and provide the best timing for surgical intervention. In order to address this issue, we proposed an DL-based decision support system for diagnosing and suggesting therapies for hip degenerative disorder. The system consisted of two main components: a deep learning algorithm for image analysis and a rule-based decision engine for recommending joint replacement. The deep learning algorithm is used to identify the severity of hip osteoarthritis from imaging data and uses convolutional neural networks to analyze the imaging data and extract important features of the hip joint. These features are then used to classify the severity of the disorder. The rule-based decision engine uses the results from the deep learning algorithm to recommend treatments. Based on the severity of hip joints, the decision engine will suggest appropriate treatment plans such as non-surgery or even surgery. The DL-based decision support system can help physicians to make suggestive decisions in the diagnosis and treatment of hip disorder. It can provide more accurate and timely recommendations, which can improve the quality of care and reduce the risks associated with the disease. Currently, the diagnosis and treatment of hip disorders rely on a combination of imaging studies, patient history, physical examination, and clinical judgment. However, DL algorithms can analyze large datasets and identify patterns that may not be visible to the human eye, potentially improving the accuracy of diagnosis and treatment recommendations. The use of DL-based decision support systems can also help to reduce the risks associated with hip disorders. delays in the diagnosis and treatment of hip disorders can lead to chronic pain, disability, and reduced quality of life. The early identification of patients who may require THR can avoid chronic pain, disability, and reduce the quality of life, and improve outcomes for patients.

To externally validate the performance of deep learning algorithms is essential. In this study, we obtained RWD to validate this algorithm to become real-world evidence (RWE) [53]. DL experienced a rapid proliferation in a wide range of RWD applications in recent years, outperforming conventional approaches [54,55,56,57]. There are numerous types of RWD. In this study, we used the health services registries-based RWD as our validation dataset and health service registries consisting of patients who had a procedure or hospitalization. It was able to share best clinical practices and support regulatory decision-making [58,59]. For small sized-clinical trials whose data are subject to high variability, often for rare disease, registries provide a valuable data source for confirmatory clinical trial design [60]. DL techniques are largely used for predictions and classification and data visualization, which may change soon as regulatory agencies are aggressively evaluating DL for generating RWE [36,61]. DL was broadly used in health informatics to generate RWE and create personalized healthcare [62,63] and was used extensively in RWD collected during the COVID-19 pandemic. It was successfully applied to understand, prevent, and assess disease [64,65]. To collect RWD is the first step. With the electronic health records improving, to gather a large and diverse dataset that includes the variables needed became easier. With the collection of RWD, it became another alternative method to validate the algorithm and, furthermore, it can be used to fine-tune the algorithm to improve the final performance and application in clinical scenarios.

One paradox in DL is the “black box” fashion and the explainable algorithm became an important issue in clinical usage of DL algorithm [39]. Therefore, visualization of the features became a solution to realize the underlying mechanism of DL algorithm. In this study, as previously experienced [52], we used visualization by the grad-CAM method to provide evidence the model indeed recognized the hip disorder. Grad-CAM is a technique used to visualize the regions of an input image that are most important for a neural network to make a particular prediction [66]. We modified the trained SurgHipNet to include a Grad-CAM layer. This layer computes the importance of each pixel in the image for the final prediction. The output of this layer consists of a heatmap that shows the ROIs that are most important for the prediction and overlaid on the input image to provide a visual representation of the important regions [67]. The heatmap indicates that the algorithm is focusing on certain parts of the hip joint; we can conclude that these features are important for predicting whether or not a patient has undergone THR. Furthermore, by comparing the heatmaps of images with and without THR, we can identify the differences in important regions between the two types of images. This can help us understand the specific features of hip joint images that are most relevant to the prediction of THR. It also can help improve the explainability of the algorithm by providing insights into how the neural network is working.

This study had some limitations. A fundamental limitation arose from the nature of DLs. In a DL, the neural network is provided only with images and associated diagnoses, without an explicit definition of features. Since DL “learned” the most predictable traits, the algorithm may be using traits previously unknown or ignored by humans. Although this study showed excellent visualization for predicting THR, the exact features used are still unknown. The results of this study are encouraging; however, the algorithms could integrate the clinical prevalence and socioeconomic status of patients to make more accurate diagnoses in the future. In this study, we could not collect the clinical parameters such as pain score and limitation of activity to train our algorithm which is another limitation of this study. To expand the data size and advanced labeling can improve the algorithm to another level. Although we collected RWD as valid material to generate RWE, the selection bias could not be completely prevented by the nature of study design. Randomized prospective studies need to be conducted to demonstrate the clinical impact of deep learning on THR prediction. Another limitation of this study was the absence of precise labeling of RWD. Therefore, we cannot offer the severity of degenerative disorder in the image of RWD, which might limit the persuasiveness of our results. Although our study included some limitations, it also presented the possibility that the DL algorithm can assist the clinical practice in different perspectives.

## 5. Conclusions

In summary, this functional deep learning algorithm can provide an accurate and reliable method for detecting hip degeneration and predicting the need for further THR, which assists physicians in decision making for surgical consultation. RWD offered an alternative support of the algorithm and validated its function to save time and cost. Additionally, the algorithm can also be used to detect progression of hip joint degeneration and provide guidance for non-surgical intervention.

## Figures and Tables

**Table 1:** The performance of SurgHipNet classification results in the testing dataset and real-world dataset.

	TP	TN	FP	FN	ACC	Sn	Sp	NPV	F1	AUC (95% CI)
2008–2017 Test data	92	372	3	8	0.977	0.920	0.992	0.979	0.944	0.994 (0.990–0.998)
2018–2019 RWD data	189	100	11	0	0.972	0.945	1.000	0.900	0.972	0.972 (0.955–0.988)

TP: true positive; TN: true negative; FP: false positive; FN: false negative; PPV: positive predictive value; NPV: negative predictive value; F1: F1 score; AUC: area under curve; CI: confidence interval.

## Data Availability

The 2008–2017 dataset and RWD dataset are not publicly available due to restrictions in the data sharing agreements with the Chang Gung Memorial Hospital Institutional Review Board (IRB). The partial dataset was available by the request to the corresponding authors under academic purpose.

## Code Availability Statement

The in-house developed codebase used here is available upon request from the authors. The hip region detection module (HipRD system) utilized PyTorch v.031 and fastai API 2018 implementation is available at https://github.com/fastai/fastai1 (accessed on 21 August 2020). The hip classification and visualization modules (SurgHipNet system) utilized PyTorch v0.4 and fastai API 2020 implementation are available at https://github.com/fastai/course-v3 (accessed on 10 May 2021 Git stats).
